# Complement–Metabolic Inflammation Crosstalk: Molecular Mechanisms and Therapeutic Targets in Type 2 Diabetes and Its Complications

**DOI:** 10.3390/cimb48101053

**Published:** 2026-10-13

**Authors:** Yue Xie, Wen Ye, Xiaohua Wen, Binbin Zhang, Jianan Zhao

**Affiliations:** 1 Artemisinin Research Center, Guangzhou University of Chinese Medicine, Guangzhou 510080, China; 2 School of Acupuncture & Moxibustion and Tuina, Tianjin University of Traditional Chinese Medicine, Tianjin 301617, China; 3 School of Clinical Medicine, Hangzhou Normal University, Hangzhou 310015, China; drzhangvip@163.com; 4 Department of Cardiovascular Sciences, Temple University, Philadelphia, PA 19122-6096, USA

**Keywords:** complement system, metabolic inflammation, type 2 diabetes, insulin resistance, diabetic complications, therapeutic targets

## Abstract

Type 2 diabetes mellitus (T2DM), a global metabolic disease, is fundamentally characterized by a vicious cycle of chronic low-grade inflammation and metabolic dysregulation. The complement system, a key effector of innate immunity, has recently been recognized as deeply involved in initiating and amplifying metabolic inflammation. Although current studies have revealed critical roles of complement components in pancreatic β-cell damage, insulin resistance, and adipose tissue inflammation, the interactive network between complement and metabolic inflammation in T2DM microvascular and macrovascular complications remains incompletely understood, and complement-targeted therapies still face specificity and safety challenges, including systemic immunosuppression risks. This review systematically summarizes the molecular crosstalk between complement components and metabolic inflammation in T2DM and its complications, focusing on complement-mediated β-cell apoptosis, suppression of insulin signaling, adipose tissue macrophage polarization, and renal and vascular endothelial injury. We also discuss the prospects of complement-targeted drug development, highlighting key candidate targets such as C3aR, C5aR1, and Factor B, as well as primary translational bottlenecks including systemic immunosuppression risks, infection susceptibility, and limited target specificity, aiming to offer new insights for precision treatment of T2DM and its complications.

## 1. Introduction

The global prevalence of type 2 diabetes mellitus (T2DM) continues to rise, representing a major public health challenge of the 21st century. Recent epidemiological data reveal a marked shift in disease burden, with prevalence escalating rapidly, particularly among younger populations (diagnosed before age 40), where early onset T2DM is associated with more aggressive disease progression, a higher burden of risk factors, and earlier, more severe complications [1]. The core pathological basis of T2DM is a chronic metabolic inflammatory state characterized by macrophage infiltration in adipose tissue, the liver, and pancreatic islets, which drives insulin resistance and β-cell dysfunction [2]. Furthermore, the interplay among inflammation, aging, and oxidative stress collectively exacerbates metabolic disturbances and insulin resistance [3].

As a cornerstone of innate immunity, the complement system has long been regarded as a key effector for pathogen clearance. However, recent studies have revealed its underappreciated regulatory role in metabolic homeostasis. Complement C3, a core component, has been consistently linked to T2DM risk: a 10-year prospective cohort study found that elevated serum C3 was independently associated with increased T2DM risk, with BMI potentially mediating approximately 41% of the effect [4]. A 7-year follow-up study demonstrated that changes in plasma C3 were positively correlated with indices of insulin resistance, and baseline C3 predicted the 7-year incidence of T2DM [5]. In patients with rheumatoid arthritis, serum C3 was also an independent predictor of T2DM comorbidity [6]. In the metabolic inflammatory environment, complement components undergo extensive local synthesis and activation, modulating inflammatory cytokine secretion, immune cell polarization, and tissue fibrosis. Plasma levels of complement C1s, C3, C9, and ficolin-3 (FCN3) are significantly upregulated in T2DM patients, with FCN3 and C9 likely intensifying inflammation via complement activation [7]. In diabetic kidney disease (DKD), the urinary abundance of complement C3, C9, complement factor H (CFAH), C5, decay-accelerating factor (DAF), and CD59 is significantly associated with the rate of renal function decline and the risk of end-stage renal disease [8]; excessive C5 activation promotes DKD progression through the STAT3 pathway and the gut–kidney axis [9]. In diabetic retinopathy, serum C3 and C5 levels are independent risk factors, positively correlated with disease duration, HbA1c, and triglyceride (TG) levels [10]. Collectively, these findings indicate that the complement system—particularly C3—has transcended its conventional immune defense functions to become an important biomarker and potential mediator of metabolic disorders and T2DM complications.

A complex bidirectional regulatory network exists between the complement system and metabolic inflammation, with critical implications for T2DM development and its complications. Metabolic inflammatory conditions (e.g., obesity, hyperglycemia) activate the complement system, while complement activation products further amplify inflammation, creating a vicious cycle. This intricate regulatory interplay indicates that the complement system is not merely a passive participant but an active regulator of metabolic inflammation.

Targeting complement components or their receptors has yielded significant improvements in preclinical models of diabetic complications, offering novel therapeutic avenues for T2DM. It should be noted that the descriptions below target complement’s role in pathological secondary complications—such as diabetic kidney disease (DKD) and retinopathy—rather than primary metabolic effects. In animal models of DKD, a C5a receptor antagonist (C5aRA) markedly improved renal function, attenuated glomerular extracellular matrix deposition, macrophage infiltration, and pro-inflammatory cytokine expression, and partially reversed the decline in gut microbiota diversity and short-chain fatty acid levels [9]. In addition, quantitative analysis of urinary complement proteins suggests that targeting complement pathways may slow DKD progression [8]. In diabetic retinopathy, serum C3 and C5 serve as predictive factors and provide potential biomarkers for the development of specific complement inhibitors [10]. These preclinical findings collectively suggest that blocking excessive complement activation may represent an effective strategy for treating T2DM and its complications.

This review aims to systematically delineate the mechanisms by which complement components participate in metabolic inflammation, clarify their relationship with T2DM and its complications, and provide a theoretical foundation for the translational application of complement-targeted therapies. To define the scope of this review precisely, we focus on the following metabolic tissues/cells and diabetic complications: (i) metabolic tissues/cells—pancreatic β-cells, adipose tissue, liver, and skeletal muscle; (ii) diabetic microvascular complications—diabetic kidney disease (DKD) and diabetic retinopathy (DR); and (iii) diabetic macrovascular complications.

### Literature Search Strategy

A comprehensive literature search was performed in PubMed and Web of Science. No publication date restriction was applied; the search was last updated on 8 September 2026. The search strategy combined Boolean operators (AND, OR) and truncation with terms related to diabetes (“diabetes mellitus,” “type 2 diabetes,” “T2DM,” “hyperglycemia”), metabolic inflammation (“metabolic inflammation,” “metabolism,” “insulin resistance,” “adipose tissue,” “pancreatic β cells,” “liver,” “skeletal muscle”), complement (“complement,” “complement system,” “C3,” “C5,” “C5a,” “C5aR1,” “Factor B,” “CD59,” “MBL,” “membrane attack complex”), and specific diabetic complications (“diabetic kidney disease,” “diabetic nephropathy,” “diabetic retinopathy,” “macrovascular disease,” “atherosclerosis,” “coronary artery disease,”). Inclusion criteria were peer-reviewed, English-language original research, reviews, meta-analyses, and clinical studies providing mechanistic, biomarker, or therapeutic evidence relevant to complement, metabolic inflammation, and T2DM and its complications. Exclusion criteria were non-English publications, conference abstracts without full data, editorials/commentaries, duplicate records, and studies not directly addressing the review topic. Titles and abstracts were screened first, followed by full-text assessment; reference lists of included articles were also manually searched to identify additional relevant studies.

## 2. Composition, Activation Pathways, and Expression of the Complement System in Metabolic Tissues

### 2.1. The Three Activation Pathways and the Common Terminal Pathway

The complement system consists of multiple components that are activated through three distinct but convergent pathways, ultimately converging on a common terminal pathway. The classical pathway is initiated by the C1 complex, in which C1q acts as a pattern recognition molecule that binds to IgG/IgM in immune complexes or damage-associated molecular patterns (DAMPs), followed by sequential activation of C1r and C1s, which cleave C4 and C2 to form the C3 convertase [11]. The lectin pathway is triggered by mannose-binding lectin (MBL) and ficolins that recognize glycosylated ligands on pathogen surfaces, forming complexes with MBL-associated serine proteases (MASP-1 and MASP-2), which also cleave C4 and C2 [12,13]. The alternative pathway is continuously activated at a low level through spontaneous hydrolysis of C3, forming C3 convertases on pathogen or damaged cell surfaces, and constitutes a powerful positive-feedback amplification loop [14]. Despite their different initiation mechanisms, all three pathways converge on a common terminal pathway: the formation of C5 convertase, which cleaves C5 into C5a and C5b. C5b sequentially assembles with C6, C7, C8, and multiple C9 molecules to form the C5b-9 membrane attack complex (MAC), which creates pores in the target cell membrane, leading to osmotic lysis [15,16]. During this process, anaphylatoxins such as C3a and C5a are released [17]. Although C5a is undoubtedly a potent pro-inflammatory mediator, C3a is best described as an “inflammatory modulator” rather than a purely pro-inflammatory factor, since it can exert anti-inflammatory functions in certain contexts [18]. Nonetheless, both anaphylatoxins contribute to immune cell recruitment and activation. Notably, MAC formation does not always result in cell death; sublytic levels of MAC can trigger intracellular signaling pathways, such as activation of the NLRP3 inflammasome and promotion of IL-1β secretion, thereby modulating the inflammatory response [19]. The activity of the complement system is tightly regulated by a series of soluble and membrane-bound regulatory proteins to prevent excessive damage to self-tissues. For example, C7 can form complexes with the inhibitory regulatory protein clusterin in the circulation, potentially affecting MAC assembly and function [20]. In recent years, an important discovery has been the existence of an intracellular complement activation system, where complement components can be activated independently of the extracellular complement system and participate in the regulation of cellular metabolism, gene expression, and inflammatory responses, providing a novel perspective for understanding the role of the complement system in metabolic diseases [21]. Of direct relevance to this review, a recent study using beta-cell-specific C3 knockout mice demonstrated that cell-intrinsic C3 is required for optimal pancreatic beta-cell function under metabolic pressure in vivo: when placed on a high-fat diet, these mice exhibited exaggerated weight gain, higher fasting glucose and insulin levels, impaired pro-insulin processing, adipose tissue inflammation, and insulin resistance [22].

### 2.2. Local Synthesis of Complement Components in Metabolic Tissues

The complement system has traditionally been viewed as a liver-derived serum protein network. However, recent studies have revealed that multiple metabolic tissues possess the capacity for local synthesis of complement components, reshaping our understanding of the complement system’s role in metabolic regulation. Adipose tissue is one of the most active sites of local complement synthesis, serving not only as a major source of C3 but also as the primary production site for factor D (adipsin) [23]. The expression of complement genes in adipose tissue is not static; rather, it undergoes significant adjustments in response to metabolic states. Under obese conditions, the complement gene expression profile in adipose tissue is markedly upregulated, particularly in white adipose tissue [24]. Locally produced complement components are not simply released into the circulation; instead, they act in a paracrine manner on neighboring adipocytes and infiltrating immune cells, forming a local complement activation network within the microenvironment [25,26]. The active fragments generated by this local complement activation, such as the anaphylatoxins C3a and C5a, can modulate the metabolic functions of adipocytes, affecting lipid storage and insulin sensitivity [27]. Notably, the role of the complement system in adipose tissue is dual: on one hand, moderate activation of complement components such as C3 and factor B contributes to normal metabolic regulation of adipose tissue. On the other hand, under obese conditions, excessive activation of the complement system may exacerbate local inflammation and promote insulin resistance [28]. Furthermore, adipose tissue-derived complement components are closely linked to systemic metabolic status. Plasma levels of C3 and C4 are positively correlated with the risk of obesity and type 2 diabetes, suggesting that local complement synthesis in adipose tissue may participate in the pathogenesis of systemic metabolic disorders by influencing circulating complement levels [28].

The liver, as the primary organ for complement synthesis, also plays an important role in metabolic diseases through its local complement expression. In non-alcoholic fatty liver disease, the expression of complement components in the liver changes significantly, with C5 being shown to promote hepatic steatosis and inflammation [29]. Studies have demonstrated that C5 plays a key role in diet-induced fatty liver models, as C5-deficient mice exhibit less hepatic fat accumulation and inflammation after high-fat diet feeding, indicating that local complement activation in the liver is involved in the pathogenesis of metabolic liver disease [29]. In addition, complement C3 expression in the liver is regulated by metabolic status, and in a hypertensive nephropathy model, upregulated local C3 expression in the kidney is closely associated with the development of proteinuria [30]. Local complement synthesis in the liver not only affects the liver’s own metabolic functions but may also influence other metabolic tissues through paracrine or endocrine mechanisms, forming an inter-tissue complement signaling network.

In diabetic nephropathy (DN), local complement activation drives renal injury. Transcriptomic analysis of DN glomeruli identified C3, C1QB, and C1QA as complement hub genes, with complement activation and humoral immune response as key pathways. Glomerular C1q and C3 deposition was associated with more severe glomerular lesions and independently predicted kidney failure, suggesting that classical complement pathway activation accelerates DN progression [31]. Skeletal muscle, as a major target tissue of insulin action, also possesses the capacity to synthesize complement components. Complement factor H and properdin are expressed in a variety of extrahepatic tissues, including skeletal muscle. This broad pattern of local synthesis suggests that the complement system may have local regulatory functions in metabolic tissues that are independent of the systemic complement system [32]. Properdin, as the only positive regulator of the alternative pathway, is expressed in adipose tissue and is closely related to lipid metabolism. Properdin-deficient mice exhibit increased body weight and fat accumulation under a high-fat diet, while supplementation with properdin suppresses insulin-mediated fatty acid uptake and triglyceride synthesis [27]. Collectively, these findings indicate that the local synthesis of complement components in metabolic tissues constitutes a complex regulatory network that participates in the maintenance of metabolic homeostasis through paracrine and autocrine mechanisms. Dysregulation of this network may promote the development and progression of type 2 diabetes and its complications.

### 2.3. Expression Patterns of Complement Regulatory Proteins in Metabolic Tissues

The expression patterns of complement regulatory proteins are key determinants of the local activation threshold and spatial distribution of the complement system in metabolic tissues. These regulatory proteins are broadly classified into membrane-bound forms (e.g., CD46, CD55, CD59) and soluble forms (e.g., factor H, C4b-binding protein, C1 inhibitor). Under normal physiological conditions, these regulators exhibit distinct expression profiles in metabolically active tissues such as adipose tissue, liver, pancreas, and skeletal muscle, collectively maintaining homeostatic balance of the complement system. For instance, podocytes, as critical components of the glomerular filtration barrier, have been shown to express multiple complement components and regulatory proteins, including C4BP, CD46, and CD59, suggesting that local complement regulatory networks play an important role in maintaining tissue microenvironment homeostasis [33]. C4BP, a major soluble complement regulatory protein with a plasma concentration of approximately 200 mg/L, effectively inhibits the formation of C3 convertases of the classical and lectin pathways by binding to C4b and serving as a cofactor for factor I, thereby controlling the complement activation cascade [34]. Moreover, C4BP is also expressed in pancreatic tissue, and its expression level can be regulated by inflammatory factors such as IL-1β, indicating that metabolic tissues possess the capacity to actively modulate local complement activation [35].

Under metabolic inflammatory conditions, the dysregulation of complement regulatory protein expression is a core mechanism leading to excessive local complement activation and exacerbated tissue damage. Obesity-associated chronic low-grade inflammation can significantly alter the expression profile of complement regulatory proteins in adipose tissue. Studies have shown that C4BP not only functions as a complement inhibitor but also exhibits a range of biological activities beyond classical complement regulation, including modulation of cell survival, influence on pathogen virulence, and participation in immune regulation [36,37]. In type 2 diabetes, aggregation of islet amyloid polypeptide (IAPP) is a key pathological feature of β-cell dysfunction. It has been found that C4BP can bind to IAPP and promote its conversion from cytotoxic oligomers to less toxic fibrillar forms, thereby protecting β-cells from IAPP-induced cytotoxicity [38,39]. However, this protective effect may be accompanied by local alterations in complement activation. Notably, different isoforms of C4BP exhibit distinct functions under inflammatory conditions. The α7β0 isoform (lacking the β-chain), which is overexpressed during the acute-phase response, can induce dendritic cells to enter a semi-mature, anti-inflammatory state, characterized by suppressed release of pro-inflammatory cytokines (e.g., IL-12, TNF-α) and increased secretion of IL-10, thereby exerting immunomodulatory effects [40]. This isoform-specific shift in expression patterns reflects the fine-tuning of the complement regulatory network by metabolic tissues under inflammatory stress.

## 3. The Role of the Complement System in Adipose Tissue Inflammation and Insulin Resistance

### 3.1. The Role of C3a/C3aR Signaling in Adipocyte Dysfunction

C3a, an anaphylatoxin generated upon complement activation, exerts a critical role in adipocyte dysfunction by binding to its specific G protein-coupled receptor, C3aR. Studies have demonstrated that the C3a/C3aR signaling axis directly modulates adipocyte metabolism and inflammatory status. In vitro, treatment of 3T3-L1 adipocytes with C3a (100 nM) exhibits insulin-like effects, promoting energy storage by increasing glucose and fatty acid uptake while inhibiting cAMP signaling and lipolysis, thereby facilitating lipid accumulation [41]. This mechanism is closely linked to the Gαi protein-coupled signaling pathway activated upon C3aR engagement, which suppresses adenylate cyclase activity, reduces intracellular cAMP levels, and consequently decreases hormone-sensitive lipase activity, ultimately inhibiting lipolysis [41].

Under obese conditions, C3aR expression in adipose tissue is markedly elevated. Animal studies have shown that in obese rats fed a high-carbohydrate, high-fat diet, C3aR and C5aR expression in visceral adipose tissue is significantly upregulated, associated with adipose tissue inflammation, glucose/insulin intolerance, and cardiovascular dysfunction [41]. Importantly, treatment with a C3aR-selective antagonist (30 mg/kg/day, oral) in obese mice significantly attenuated weight gain, reduced visceral fat accumulation, improved adipose tissue insulin sensitivity, and lowered pro-inflammatory adipokine levels [41]. Further investigations revealed that C3aR antagonist treatment also reduced macrophage infiltration into adipose tissue and altered the expression profile of inflammation-related genes, indicating that C3a/C3aR signaling not only directly affects adipocyte function but also participates in shaping the inflammatory microenvironment of adipose tissue by regulating immune cell recruitment and activation [41]. The activation mechanism of C3aR involves ligand-induced conformational changes; endogenous peptide ligands such as TLQP-21 bind to C3aR via a “fold-and-bind” mechanism, with specific C-terminal amino acid residues (e.g., R21) being critical for receptor activation [42].

Moreover, C3aR is expressed on multiple cell types, including adipocytes, macrophages, and mast cells, and its signaling exhibits cell-type specificity. In macrophages, C3a induces the release of prostaglandin E2 (PGE2), further amplifying the inflammatory response [41]. In mast cells, C3aR activation requires scaffold proteins such as NHERF1 and NHERF2, which, although not directly binding to C3aR, modulate downstream signaling pathways (e.g., NF-κB) to influence C3a-induced degranulation and chemokine production [43]. These findings collectively reveal the multifaceted mechanisms of C3a/C3aR signaling in adipocyte dysfunction, including direct promotion of lipid accumulation, induction of pro-inflammatory cytokine secretion, suppression of insulin signaling, and modulation of immune cell function, providing a strong rationale for developing C3aR-targeted therapeutic strategies for metabolic diseases.

### 3.2. C5a/C5aR-Mediated Macrophage Polarization and Adipose Tissue Inflammation

The binding of C5a to C5aR1 plays a pivotal role in regulating macrophage polarization, promoting a shift toward the pro-inflammatory M1 phenotype while suppressing the anti-inflammatory M2 phenotype, thereby exacerbating adipose tissue inflammation. Studies have shown that C5a activates macrophages through its receptor C5aR1 (CD88), enhancing their phagocytic activity and the release of inflammatory cytokines such as TNF-α and IL-6 [44]. In obesity and T2DM, elevated C5a levels in adipose tissue drive macrophage polarization toward the M1 phenotype, accompanied by a significant reduction in the proportion of M2 macrophages [45]. This polarization imbalance leads to chronic low-grade inflammation in adipose tissue, which in turn promotes the development of insulin resistance. Furthermore, activation of C5aR1 also promotes the release of IL-1β and HMGB1 via the NLRP3 inflammasome pathway, further amplifying the inflammatory response [46]. Additionally, C5aR1 expression on vascular endothelial cells is involved in regulating angiogenesis and inflammatory responses, suggesting a broad role in systemic metabolic inflammation [47].

However, C5a signaling is not mediated by C5aR1 alone. As the second C5a receptor, C5aR2 initiates its own signaling and exerts notable immunoregulatory effects, although its function relative to C5aR1 remains debated and context-dependent [48]. In adipocyte biology, C5aR2 (also known as C5L2) serves as the receptor for acylation-stimulating protein (ASP), an adipocyte-derived adipokine that promotes triglyceride synthesis and storage and enhances glucose and fatty acid uptake in adipose tissue [49]. Therefore, the ASP-C5L2 interaction is considered a potential pharmacological target [49]. Consistent with this metabolic role, C5L2 polymorphisms (e.g., rs2972607) are associated with HDL-C, glucose, and inflammatory markers [50], supporting the importance of C5aR2 in lipid metabolism, insulin sensitivity, and metabolic regulation. Supporting a protective role for C5aR2, properdin deficiency in high-fat-diet mice increased hepatic triglyceride content and steatohepatitis and reduced C5L2 (C5aR2) expression in adipose membranes and circulating microparticles [51]. These findings suggest that C5aR2 protects against obesity-related metabolic inflammation and that its downregulation exacerbates this condition. Mechanistically, C5aR2 activation suppresses cytokine production triggered by TLR2, TLR3, TLR4, and TLR7, as well as C-type lectin receptor-mediated IL-6 and TNF-α production, and inhibits ERK signaling downstream of C5aR1, C3aR, and chemokine-like receptor 1 [52]. C5aR2 therefore exerts broad anti-inflammatory effects through decoy activity and signal cross-inhibition. In breast cancer, C5AR2 expression correlates with immune infiltration, particularly macrophage polarization [53], further supporting a context-dependent role in shaping macrophage phenotypes.

Collectively, C5aR1 and C5aR2 may form a regulatory balance in adipose tissue: C5aR1 primarily drives M1 polarization and metabolic inflammation, whereas C5aR2 restrains excessive complement activation and inflammation while exerting context-dependent effects on lipid handling and adipocyte biology. Targeting the C5a–C5aR1 axis while accounting for C5aR2-mediated regulation may therefore interrupt the inflammatory positive-feedback loop in T2DM and its complications without compromising C5aR2-mediated regulatory effects.

### 3.3. Local Complement Activation in Adipose Tissue and Its Interplay with Lipid Metabolism

Adipose tissue is not only a core organ for energy storage but also a key site where local complement activation intersects with metabolic regulation. Studies have shown that in obesity, the expression of complement genes in adipose tissue undergoes significant changes: components of the early complement pathway (e.g., C1q, C1s, C2) are upregulated, while terminal pathway components (e.g., C5, C6) are downregulated. This imbalanced complement activation profile is closely associated with adipose tissue inflammation and insulin resistance [54].

Adipose tissue-derived adipsin (factor D) is upregulated in obesity and participates in adipose tissue remodeling and fibrosis by promoting alternative pathway activation. Adipsin is a serine protease synthesized by adipocytes; its precursor form must be cleaved by MASP-3 to the mature form in order to cleave the complex of factor B and C3b and form the C3 convertase (C3bBb), a core step in alternative pathway activation and amplification [55,56]. In obese individuals, upregulated adipsin expression in adipose tissue leads to excessive alternative pathway activation, generating large amounts of anaphylatoxins such as C3a and C5a, which recruit immune cells and promote adipose tissue fibrosis. Notably, adipsin activity is tightly regulated; the ratio of precursor to mature form in the circulation is approximately 8–15%, and this balance may be disrupted in obesity, resulting in sustained complement activation [55]. Furthermore, in obese mouse models, C5 deficiency exacerbates glucose intolerance in male mice, suggesting a sex-dependent role of the terminal complement pathway in adipose tissue metabolic regulation [57]. However, since C5 deficiency prevents both MAC formation and C5aR signaling, the observed effects cannot be attributed exclusively to MAC; C5aR-mediated signaling may also contribute to the metabolic phenotype. These findings collectively reveal a complex network through which the complement system regulates adipocyte differentiation and lipid metabolism at multiple levels, providing new insights into the molecular mechanisms of metabolic inflammation.

## 4. Mechanisms of the Complement System in Pancreatic β-Cell Injury and Dysfunction

### 4.1. Complement Deposition in the Islet and β-Cell Injury

Local complement activation persists within pancreatic islets. Studies have confirmed that the aggregation and fibrillation of islet amyloid polypeptide (IAPP) are key factors in β-cell death in T2DM. IAPP fibrils can directly bind to the globular head domain of the complement initiation molecule C1q, triggering limited activation of the classical pathway, and C3d deposition has been observed in vitro [39]. Furthermore, immunohistochemical staining of pancreatic sections from T2DM patients reveals the presence of complement components within islets, with varying degrees of colocalization between IAPP fibrils and C1q or C3d, further confirming complement activation in the islet microenvironment [39]. This local complement deposition is not limited to IAPP-related pathology. In human islet transplantation, the early deposition of complement onto transplanted islets is a well-documented phenomenon known as the “instant blood-mediated inflammatory reaction (IBMIR)” [58], which represents a clear case of complement activation triggered by human pancreatic islets. Similarly, in xenotransplantation models, porcine islet cells exhibit C3 deposition early after transplantation, whereas supporting cells with immune-privileged properties effectively inhibit C3 and membrane attack complex (MAC) deposition by highly expressing complement regulatory proteins such as CD46 and CD55, indirectly reflecting the susceptibility of islet cells to complement-mediated injury [59].

The mechanisms of complement-mediated β-cell injury are complex, involving both direct cell lysis induced by C5b-9 and the triggering of apoptosis and dysfunction at sublytic doses. When a sufficient number of C5b-9 complexes are inserted into the cell membrane, they form transmembrane pores, leading to osmotic lysis and necrotic cell death, a process accompanied by elevated intracellular calcium levels and activation of death signals such as JNK, RIPK1, and RIPK3 [60]. However, at sublytic doses, C5b-9 deposition does not immediately cause cell death but instead induces cell dysfunction through the activation of multiple signaling pathways. Studies have shown that in Barrett’s esophagus and esophageal adenocarcinoma cell lines, exposure to sublytic doses of C9 leads to C5b-9 complex formation, which is subsequently internalized and shed from the cell surface via extracellular vesicles, allowing cells to evade complement-mediated killing. However, this process may be accompanied by alterations in cell phenotype and function [61]. In pancreatic β-cells, similar sublytic C5b-9 deposition may activate signaling pathways such as MAPK, inducing endoplasmic reticulum stress and mitochondrial dysfunction, ultimately impairing insulin secretion and promoting apoptosis [62]. Additionally, the anaphylatoxins C3a and C5a generated by complement activation can bind to their respective receptors on the β-cell surface, exacerbating inflammatory responses and cell injury [63]. However, C3aR signaling can also augment insulin secretion [64]. Thus, complement anaphylatoxins exert a dual role in β-cell function, and their net effect likely depends on the specific context of complement activation.

### 4.2. Potential Roles of Complement Regulatory Proteins in Islet Protection

Excessive activation of the complement system is a major contributor to β-cell injury, and complement regulatory proteins play a critical role in maintaining complement homeostasis and protecting β-cells from attack. CD59 is a widely expressed glycosylphosphatidylinositol (GPI)-anchored protein whose primary function is to inhibit the formation of the membrane attack complex (MAC), thereby protecting host cells from complement-mediated lysis. In pancreatic β-cells, CD59 expression levels are remarkably high, suggesting a particular importance in β-cell protection [65]. Further studies have revealed that the role of CD59 in β-cells extends beyond complement inhibition. In addition to the classical membrane-bound form, human pancreatic β-cells also contain non-GPI-anchored intracellular CD59 isoforms generated through alternative splicing, namely IRIS-1 and IRIS-2 (Isoforms Rescuing Insulin Secretion 1 and 2) [66]. These intracellular isoforms interact with SNARE proteins (e.g., VAMP2 and SNAP25) and directly participate in the exocytosis of insulin secretory granules, which is essential for normal insulin secretion [65,66]. Notably, the expression levels of IRIS-1 and IRIS-2 are significantly reduced in the islets of type 2 diabetes (T2D) patients, and high-glucose conditions (glucotoxicity) also lead to decreased IRIS-1 expression in primary human islets, suggesting that the downregulation of CD59 and its isoforms may be closely linked to β-cell dysfunction and impaired insulin secretion in T2D [66]. Therefore, CD59 not only provides protection by inhibiting MAC formation but also directly regulates insulin secretion through its intracellular isoforms, and maintaining its expression level is crucial for β-cell function.

**Figure 1:**
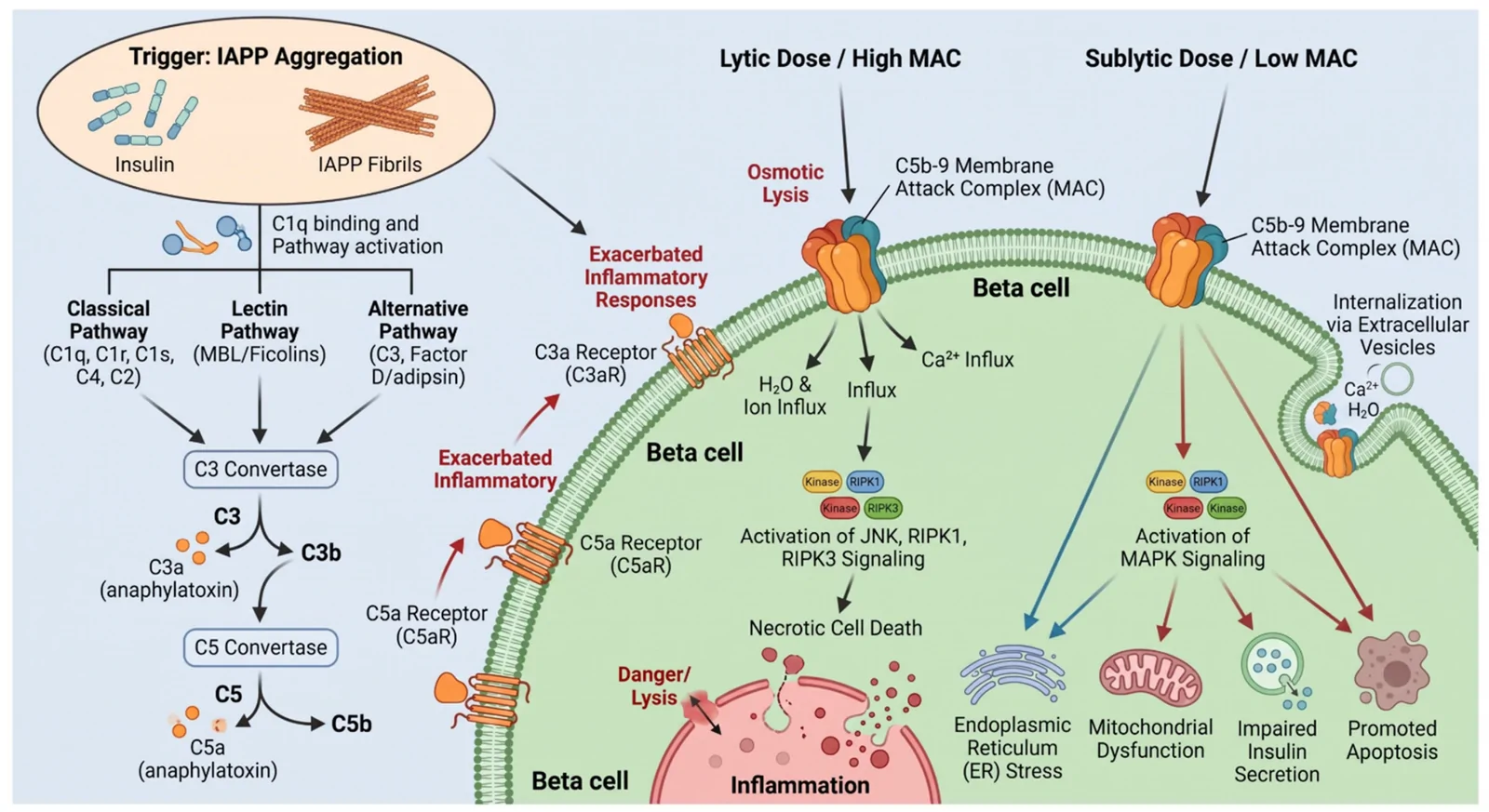
Complement-mediated pancreatic β-cell injury and dysfunction in T2DM. IAPP aggregation promotes complement activation and generation of C3a, C5a, and C5b-9. High levels of MAC induce membrane pore formation, calcium influx, and necrotic cell death, whereas sublytic MAC activates MAPK-related signaling, ER stress, mitochondrial dysfunction, impaired insulin secretion, and apoptosis. C3a/C3aR and C5a/C5aR signaling further amplify local inflammatory responses. Abbreviations: C3aR, C3a receptor; C5aR, C5a receptor; IAPP, islet amyloid polypeptide (amylin); MAC, membrane attack complex (C5b-9); MBL, mannose-binding lectin. Created in BioRender. Zhao, J. (2026) https://BioRender.com/te0kgjc.

Given the critical roles of complement regulatory proteins in islet protection, strategies such as exogenous supplementation or enhancement of endogenous expression to increase their levels have emerged as potential therapeutic approaches to protect β-cells from complement attack. C4b-binding protein (C4BP) is a major soluble inhibitor of the classical and lectin pathways, acting by binding to C4b and serving as a cofactor for factor I to inhibit C3 convertase formation [67]. In the islet, C4BP exhibits multiple protective functions. First, C4BP can bind to IAPP, inhibiting IAPP-induced NLRP3 inflammasome activation and IL-1β production, thereby reducing IAPP-mediated β-cell cytotoxicity [35]. Second, C4BP directly protects β-cells from IAPP-induced cytotoxicity through mechanisms that include promoting IAPP fibril formation to reduce the concentration of toxic oligomers and, after internalization, protecting cells by enhancing cholesterol synthesis [38]. Additionally, C4BP inhibits IAPP-induced erythrocyte lysis [38,39]. These findings indicate that C4BP is a multifunctional protective protein. Therefore, exogenous supplementation of soluble C4BP, or overexpression of membrane-bound regulatory proteins such as CD59 in β-cells via gene therapy, may represent effective methods to enhance β-cell resistance to complement attack and delay diabetes progression. In addition, pre-treatment of islets with soluble complement inhibitors has been shown to protect them from complement attack in transplantation settings; for example, soluble complement receptor 1 (CR1) has been used to coat islets in xenotransplantation, protecting them from complement activation [68]. Furthermore, the use of bispecific antibodies to recruit endogenous C4BP or factor H to specific cell surfaces to achieve localized, targeted complement inhibition is an emerging therapeutic concept [69]. These strategies provide important directions for the development of novel complement-based therapies for diabetes (Figure 1).

## 5. Complement Signaling Crosstalk in Insulin Target Tissues

### 5.1. Hepatic Complement Expression and Regulation of Glucose and Lipid Metabolism

As the primary organ for the synthesis of circulating complement proteins, the liver undergoes significant changes in complement expression and function under type 2 diabetes (T2DM), profoundly affecting systemic glucose and lipid homeostasis. Studies have shown that in T2DM and related metabolic liver diseases, the capacity of hepatocytes to synthesize key complement components such as C3, C4, and factor B is markedly enhanced, leading to elevated circulating complement levels. This not only serves as a marker of systemic inflammation but also directly contributes to the dysregulation of metabolic networks [70]. For example, in patients with non-alcoholic steatohepatitis (NASH), the activation status of hepatic C3 (measured by the ratio of C3c to native C3) is significantly increased and positively correlated with liver inflammation scores, suggesting that local complement activation in the liver is a key driver of metabolic inflammation progression [70]. Furthermore, studies on alcoholic liver disease (ALD) have confirmed that ethanol exposure activates the hepatic complement system, resulting in markedly enhanced immunoreactivity of C1q, C3, and C5, which further exacerbates liver injury [71]. These findings collectively reveal the ubiquity of hepatic complement upregulation under metabolic stress and its pathological significance.

### 5.2. Effects of Complement Signaling on Glucose Uptake in Skeletal Muscle

Skeletal muscle is the primary target organ for glucose metabolism and insulin action, and its normal glucose uptake capacity is essential for maintaining systemic glucose homeostasis. Skeletal muscle cells express multiple complement receptors, including C3aR and C5aR, which are key molecules through which complement activation products such as C3a and C5a exert their biological effects. The tyrosine phosphorylation of insulin receptor substrate-1 (IRS-1) is a key initiating event in insulin signal transduction, and its functional status directly determines downstream PI3K/Akt activation. Recent evidence indicates that complement activation products can directly suppress IRS-1 tyrosine phosphorylation. In differentiated human skeletal muscle cells, C5a binding to C5aR1 significantly inhibited insulin-induced association of IRS1 with the PI3K p85 subunit, decreased Akt phosphorylation by approximately 50%, and reduced insulin-stimulated glucose uptake by approximately 60% [72]. Consistent with the negative regulation of IRS-1 by serine phosphorylation, IRS-1 Ser312 phosphorylation was elevated and IRS-2 expression was reduced in visceral adipose tissue from obese women with gestational diabetes, suggesting that a complement-related inflammatory environment may impair IRS-1 tyrosine phosphorylation through serine phosphorylation [73]. Quantitative phosphoproteomics further mapped 33 phosphorylation sites on skeletal muscle IRS1 and showed that insulin dynamically regulates multiple serine/threonine sites, indicating that the IRS-1 phosphorylation network is highly complex under both physiological and pathological conditions [74]. In skeletal muscle atrophy models, activation of complement and coagulation cascades was associated with upregulation of protease inhibitors such as Serpina3 and Serpina1b, factors linked to diabetes and cardiovascular disease risk [75]. Notably, ATG-125 prevented sucrose-induced skeletal muscle atrophy by upregulating the IGF-1R–IRS–PI3K–AKT pathway and inhibiting E3 ubiquitin ligases such as atrogin1 and TRIM63, suggesting that the balance between complement signaling and the ubiquitin–proteasome system is important for IRS-1 homeostasis [75]. Collectively, these findings indicate that complement signaling can directly inhibit the IRS-1–PI3K–Akt axis in skeletal muscle, thereby reducing insulin-stimulated glucose uptake, which depends on GLUT4 translocation.

Under pathological conditions such as metabolic inflammation, when the complement system is overactivated, anaphylatoxins like C5a bind to C5aR on skeletal muscle cells, subsequently activating downstream inflammatory signaling pathways [76]. Moreover, the association between the complement system and skeletal muscle pathology has been confirmed in neuromuscular diseases such as amyotrophic lateral sclerosis (ALS). In the tibialis anterior muscle of the hSOD1G93A mouse model, the expression levels of C1qB, C3, C5a, and C5aR1 increase with disease progression, and the C5a-C5aR1 signaling axis drives macrophage infiltration into skeletal muscle, further exacerbating neuromuscular junction denervation and muscle dysfunction [76]. In cardiac and skeletal muscle pathology, complement activation is considered a double-edged sword: under conditions such as ischemia, complement is recruited by damaged cells, and excessive activation exacerbates tissue injury, whereas moderate complement activation may be involved in waste clearance and regeneration processes [77]. Importantly, skeletal muscle tissue itself is also a significant source of complement components. A novel serum protein, MAP-1, encoded by the MASP1 gene, is highly expressed in cardiac and skeletal muscle tissues. This protein binds to MBL and ficolins, inhibiting complement activation, suggesting the existence of a fine-tuned complement regulatory network within skeletal muscle [78].

Exercise training is an effective non-pharmacological intervention for improving insulin sensitivity, and its mechanism may partially involve the regulation of the complement system in skeletal muscle. Studies have shown that regular physical activity significantly influences the activity status of the complement system, thereby improving the metabolic inflammatory environment. During exercise, the metabolic microenvironment of skeletal muscle undergoes dramatic changes, including energy metabolism shifts, local hypoxia, and mechanical stress, all of which may trigger complement activation [79]. However, long-term regular exercise training appears to induce an adaptive immune-metabolic response, reducing the expression levels of pro-inflammatory complement components in skeletal muscle. For example, in obesity and T2DM models, the expression of complement components such as acylation-stimulating protein (ASP, C3adesArg) is typically elevated in adipose tissue and skeletal muscle, whereas exercise training may indirectly downregulate complement gene expression in skeletal muscle by reducing adipose tissue inflammation and improving systemic metabolic status [44]. ASP itself has pro-inflammatory effects, inducing the production of cytokines such as IL-6 and TNF-α and promoting M1 macrophage infiltration, which in turn exacerbates sensitization to insulin in skeletal muscle [44]. By reducing levels of complement components such as ASP, exercise training may help break this vicious cycle between inflammation and insulin resistance. Additionally, exercise enhances the expression of complement regulatory proteins in skeletal muscle, such as MAP-1, which is highly expressed in skeletal muscle and effectively inhibits excessive complement activation [78]. This regulatory effect helps maintain the normal functions of the complement system in tissue repair and immune defense while preventing its overactivation from impairing insulin signaling. From a broader immunometabolic perspective, the regulation of the complement system by exercise training is a key component of its effect on improving systemic metabolic health. By reducing levels of central complement components such as C3, exercise alleviates the chronic low-grade inflammation associated with insulin resistance, metabolic syndrome, and T2DM [80]. Therefore, exercise training, by downregulating the expression of pro-inflammatory complement genes in skeletal muscle and enhancing complement regulatory mechanisms, may represent a key molecular mechanism underlying its improvement of skeletal muscle insulin sensitivity and glucose uptake capacity, providing an important theoretical basis for lifestyle interventions in the prevention and treatment of T2DM.

## 6. Complement System and Diabetic Nephropathy: From Inflammation to Fibrosis

### 6.1. Glomerular Complement Deposition and Podocyte Injury

In the early stages of diabetic nephropathy (DN), significant complement deposition is detectable within the glomerulus, a phenomenon closely associated with disease progression. Clinicopathological studies have confirmed that in renal biopsy tissues of DN patients, deposition of complement activation products such as C3 and C5b-9 is widespread in the glomerular mesangium and basement membrane, and the intensity of deposition is positively correlated with the level of proteinuria [81,82]. Moreover, C3c deposition is not only a marker of DN but also an independent risk factor for poor renal prognosis; patients with positive C3c deposition have a significantly higher risk of progression to end-stage renal disease [82,83]. These findings indicate that the complement system plays a central role in the pathogenesis of DN, with the degree of deposition directly reflecting the severity of glomerular injury.

Direct injury to podocytes by complement activation products is a key mechanism disrupting the glomerular filtration barrier. As terminally differentiated cells, podocytes are particularly susceptible to complement-mediated injury. Studies have shown that deposition of C5b-9 can directly induce podocyte apoptosis and foot process effacement, compromising the structural integrity of the glomerular filtration barrier [84]. In experimental models, increased complement deposition is closely associated with upregulation of podocyte injury markers such as desmin, accompanied by downregulation of slit diaphragm proteins including nephrin and podocin [85,86]. For example, in MYH9-related disorders, segmental foot process effacement is a common pathological feature, often accompanied by immunoglobulin and complement deposition [87]. Furthermore, in podocyte infolding glomerulopathy, dysregulation of the podocyte cytoskeletal protein α-actinin-4 (ACTN4) has been shown to participate in podocyte injury together with complement deposition [88]. These findings collectively demonstrate that complement activation products directly attack podocytes, leading to structural and functional abnormalities, thereby disrupting the filtration barrier and causing proteinuria.

The hyperglycemic environment is a key driver of excessive local complement activation in the glomerulus. Under diabetic conditions, hyperglycemia can directly upregulate the expression of complement receptors C3aR and C5aR on glomerular cells, thereby enhancing local sensitivity to complement signals and forming a vicious cycle [84]. Simultaneously, high glucose conditions can induce downregulation of complement regulatory proteins such as CD55/DAF on the surface of glomerular endothelial cells and podocytes, weakening the cell’s natural defense against complement activation [89,90]. For instance, endothelial-specific deletion of Krüppel-like factor 4 (KLF4) leads to reduced CD55 expression, thereby exacerbating glomerular complement deposition [90]. In addition, deposition of mannose-binding lectin (MBL) is significantly increased in diabetic glomeruli, suggesting that activation of the lectin pathway also participates in this process [91,92]. These findings collectively indicate that the hyperglycemic environment, by upregulating complement receptors and downregulating complement regulatory proteins, places the glomerulus in a state of complement susceptibility, thereby amplifying complement-mediated podocyte injury and accelerating DN progression.

### 6.2. The Role of C3a/C5a Signaling in Tubulointerstitial Inflammation

In chronic kidney diseases such as diabetic nephropathy, tubulointerstitial inflammation and fibrosis are key pathological processes leading to progressive loss of renal function. Proteinuria, as a hallmark of glomerular injury, not only reflects disruption of the filtration barrier but also acts as a pathogenic factor directly stimulating tubular epithelial cells. Studies have shown that under stress conditions such as proteinuria, tubular epithelial cells can express and secrete various complement components, including C3 and C5, thereby initiating a local complement activation cascade in the tubulointerstitium [93]. The locally produced complement activation products, particularly the anaphylatoxins C3a and C5a, bind to their respective receptors on tubular epithelial cells and infiltrating immune cells, playing a central role in amplifying interstitial inflammation and driving fibrosis [94].

C3a and C5a, by activating their specific receptors C3aR and C5aR1, can significantly promote epithelial–mesenchymal transition (EMT) in tubular epithelial cells. In vitro experiments have confirmed that exposure of the human renal tubular epithelial cell line HK-2 to C3a or C5a induces fibroblast-like morphological changes, loss of microvilli, upregulation of α-smooth muscle actin (α-SMA) and type I collagen (Col-I), and downregulation of the epithelial marker E-cadherin. These typical EMT features depend on activation of the transforming growth factor-β1 (TGF-β1)/connective tissue growth factor (CTGF) signaling pathway [95]. Further studies have revealed that C3a and C5a can also induce expression of β-catenin, a key transcriptional coactivator downstream of the TGF-β/Smad signaling pathway, whose upregulation promotes transcription of EMT-related genes [96]. In vivo, using a Crry (a C3 convertase inhibitor)-deficient mouse model, transplantation of Crry-/- C3-/- kidneys into C3aR- or C5aR-deficient recipients revealed that C3aR deficiency significantly reduced the infiltration of 7/4+F4/80+CD11b+ myelomonocytic cells and Thy1.2+ T cells in the tubulointerstitium, and attenuated interstitial deposition of collagen I and III, thereby preserving renal function; in contrast, C5aR deficiency did not show similar protective effects, suggesting that C3a and C5a play distinct but synergistic roles in tubulointerstitial injury [97].

Furthermore, C3a/C5a signaling exacerbates interstitial inflammation by regulating chemokine expression. Complement activation upregulates the expression of chemokines such as monocyte chemoattractant protein-1 (MCP-1) and regulated upon activation, normal T cell expressed and secreted (RANTES) in renal tissue, thereby promoting monocyte/macrophage recruitment to the renal interstitium [93]. In patients with IgA nephropathy (IgAN), increased glomerular deposition of C3b/C5b and increased glomerular expression of the anaphylatoxin receptors C3aR/C5aR are significantly associated with lower baseline eGFR, higher 24-h proteinuria, and a faster rate of eGFR decline. Moreover, increased deposition of C3b/C5b and increased expression of C3aR/C5aR can accelerate progression from advanced IgAN to end-stage renal disease [98]. In a respiratory syncytial virus (RSV)-induced IgAN mouse model, activation of the C5a-C5aR1 axis led to increased frequencies of Th1 and Th17 cells and decreased Treg cell frequencies, aggravating renal injury, while blockade of C5aR1 attenuated this immune imbalance and renal damage [99]. These findings collectively indicate that C3a/C5a signaling, by directly inducing EMT in tubular epithelial cells, promoting extracellular matrix deposition, and recruiting inflammatory cells, constitutes an important driving mechanism of tubulointerstitial inflammation and fibrosis. Targeting this signaling pathway may offer new therapeutic strategies for diseases such as diabetic nephropathy.

### 6.3. Experimental Progress of Complement-Targeted Therapy in Diabetic Nephropathy

Targeted therapies against the complement system have achieved significant progress in experimental studies of diabetic nephropathy (DN), with C5aR1 antagonists showing promising therapeutic potential. Studies have demonstrated that the C5a/C5aR signaling pathway plays a key role in the pathogenesis of DN. In the db/db mouse model, treatment with a novel C5a inhibitor NOX-D21 significantly reduced serum triglyceride levels and decreased the expression of diacylglycerol acyltransferase-1 and sterol regulatory element-binding protein-1 in the kidney, thereby reducing lipid accumulation [100]. Moreover, NOX-D21 treatment lowered serum urea nitrogen and creatinine levels, attenuated glomerular and tubulointerstitial injury, and reduced the expression of transforming growth factor-β1 (TGF-β1) by inhibiting the PI3K/Akt signaling pathway, thereby ameliorating tubulointerstitial fibrosis [100]. Another study also confirmed that a C5aR antagonist (C5aRA) alleviated renal dysfunction, extracellular matrix deposition, macrophage infiltration, and pro-inflammatory factor expression in db/db mice, with the mechanism possibly involving inhibition of the STAT3 pathway and improvement of the gut–kidney axis function [9]. These results collectively indicate that blocking C5aR signaling is an effective strategy for intervening in DN progression.

Anti-C5 monoclonal antibodies (e.g., eculizumab analogs) have also demonstrated renal protective effects in experimental DN models. Studies have found that C5a expression levels are significantly elevated in renal biopsy tissues of diabetic nephropathy patients, and the intensity is positively correlated with disease progression [100]. By using C5 inhibitors such as eculizumab, C5 cleavage can be blocked, thereby inhibiting the generation of downstream C5a and C5b-9. The membrane attack complex, by causing cell injury and tissue inflammation in various kidney diseases, leads to sclerosis and fibrosis [101]. In diabetic nephropathy, C5b-9 deposition is closely associated with tubulointerstitial injury, and inhibiting its formation can attenuate renal inflammation and fibrotic processes [102].

Factor B (CFB) inhibitors, by selectively blocking the activation of the alternative complement pathway, reduce renal injury while preserving the immune function of the classical pathway, exhibiting superior safety potential. Excessive activation of the alternative pathway is a major feature of complement dysregulation in DN. Studies have found that in diabetic nephropathy patients, staining for Bb, CFB, C3a, C5a, and C5b-9 is significantly enhanced in the tubulointerstitium [103]. In Cfb knockout diabetic mice, tubulointerstitial injury was significantly attenuated, and ceramide biosynthesis was inhibited [103]. Mechanistically, CFB deficiency suppressed activation of the NF-κB signaling pathway, thereby blocking the transcription of ceramide synthase, reducing ceramide synthesis, and protecting tubular cells from high glucose-induced endoplasmic reticulum stress, oxidative stress, and apoptosis [103]. Furthermore, a glomerular proteomics study in DN patients confirmed that alternative pathway-related proteins (e.g., CFB, CFH, etc.) are significantly upregulated in DN glomeruli and are positively correlated with glomerular pathological grade and proteinuria [104]. Therefore, targeting CFB to specifically inhibit the alternative pathway not only effectively alleviates renal injury in DN but also avoids comprehensive inhibition of the classical and lectin pathways, thereby reducing infection risk, representing a highly promising therapeutic strategy.

## 7. Complement System Involvement in Diabetic Retinopathy and Cardiovascular Complications

In the pathological progression of diabetic retinopathy (DR), aberrant activation of the complement system may be a key factor leading to retinal microvascular injury and disruption of the blood–retinal barrier (BRB). Clinical studies have confirmed the importance of complement activation in age-related macular degeneration (AMD), particularly in relation to the formation of membrane attack complexes in aging choriocapillaris [105]. This local accumulation of complement components directly triggers immune attack against retinal microvascular endothelial cells. However, compared with AMD, direct evidence for complement dysregulation in DR remains relatively limited. The specific triggers, regulatory mechanisms, and temporal sequence of complement activation in the diabetic retina are still poorly understood. It also remains unclear whether complement inhibition can safely and effectively prevent or slow DR progression. Therefore, further mechanistic and clinical studies—including longitudinal human studies and randomized controlled trials—are needed to clarify the role of complement in DR, identify reliable biomarkers and therapeutic targets, and determine which patients may benefit from complement-targeted therapy.

Furthermore, the complement system plays a critical role in the development of diabetic macroangiopathy and atherosclerosis. A large body of evidence has confirmed that complement components C3 and C5 are abundantly deposited in atherosclerotic plaques, and their activation products promote foam cell formation and plaque instability through multiple mechanisms. In patients with T2DM, elevated circulating C3 levels are closely associated with insulin resistance and cardiovascular disease risk, and this association is partially mediated by hepatic steatosis [106]. Moreover, C3 has been identified as a novel risk factor, and in the Atherosclerosis Risk in Communities (ARIC) study, it was shown to modestly improve risk prediction models for T2DM [107]. Within atherosclerotic plaques, the complement system is activated via the classical, lectin, and alternative pathways. Cholesterol crystals can be recognized by the lectin and classical pathways, leading to activation of C3 and C5 and the release of the potent inflammatory mediator C5a [108]. This complement activation interacts with the NLRP3 inflammasome–caspase-1 pathway, promoting IL-1β production and thereby exacerbating intraplaque inflammation [108]. Proteomic analysis of early atherosclerotic plaques has shown that complement system components are among the significantly upregulated proteins in early plaques, with C5 protein levels markedly increased in the intima of fibrolipid plaques and associated with in situ complement activation [109]. In clinical cohorts, plasma C5 levels are independently associated with the presence of systemic subclinical atherosclerosis and positively correlated with total plaque volume and coronary artery calcification, suggesting that C5 may serve as a novel circulating biomarker for subclinical atherosclerosis [109].

The complement system accelerates the progression of atherosclerosis by modulating the expression of adhesion molecules on endothelial cells, thereby promoting monocyte migration into the subendothelial space. Under diabetic conditions, hyperglycemia and oxidative stress activate vascular endothelial cells, upregulating the expression of intercellular adhesion molecule-1 (ICAM-1) and vascular cell adhesion molecule-1 (VCAM-1). Complement activation products, particularly C5a, further enhance the expression of these adhesion molecules on endothelial cells, promoting monocyte adhesion and transendothelial migration [110]. In experimental diabetic models, the assembly of the terminal complement complex (TCC) has been shown to be involved in early vascular inflammation and morphological changes. In C6-deficient rats, after short-term diabetes, the expression of VCAM-1 in mesenteric vessels, as well as leukocyte adhesion and extravasation, was significantly reduced [110]. In addition, deficiency of the complement regulatory protein CD59 accelerates diabetes-induced atherosclerosis. In ApoE-/- diabetic mice, CD59 deficiency led to increased aortic root plaque area, greater lipid deposition, enhanced MAC deposition, and increased macrophage infiltration [111]. Importantly, CD59 is susceptible to non-enzymatic glycation under hyperglycemic conditions. Work by Halperin and colleagues has shown that glycation occurs on human CD59 but not on mouse CD59; glycated human CD59 exhibits impaired complement-regulatory function, thereby promoting MAC deposition and complement-mediated endothelial injury [112]. This species-specific difference suggests that mouse models may underestimate the contribution of CD59 glycation to complement dysregulation in human diabetes. Therefore, glycation-induced inactivation of CD59 represents an overlooked mechanism of systemic complement dysregulation and inflammation in human hyperglycemia. This indicates that in the diabetic environment, dysregulation of complement regulatory mechanisms, including glycation-induced inactivation of human CD59, exacerbates complement-mediated vascular injury. Notably, mannose-binding lectin (MBL) shows a biphasic association with carotid intima-media thickness (cIMT) in T2DM patients, with both excessively high and low MBL levels being associated with increased cIMT, suggesting that MBL may have a complex dual role in diabetic macroangiopathy [113].

**Figure 2:**
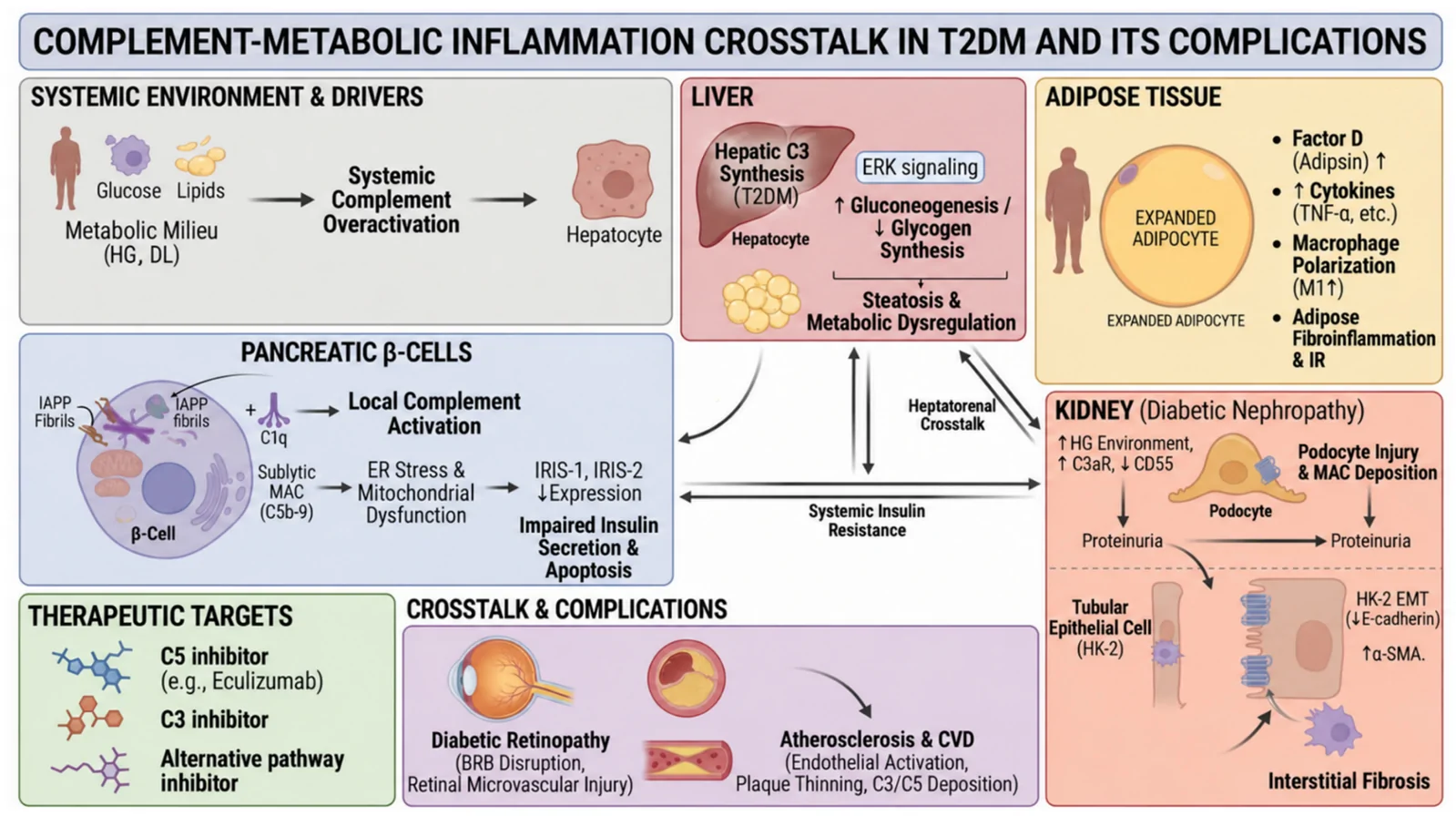
Complement–metabolic inflammation crosstalk in T2DM and its complications. Metabolic stress promotes systemic and tissue-specific complement activation, which contributes to pancreatic β-cell dysfunction, hepatic metabolic dysregulation, adipose tissue inflammation and insulin resistance, and diabetic kidney injury. Complement-mediated inflammatory signaling also participates in diabetic retinopathy and cardiovascular complications, highlighting potential therapeutic targets including C3, C5, and alternative pathway components. Abbreviations: BRB, blood–retinal barrier; CVD, cardiovascular disease; DL, dyslipidemia; HG, hyperglycemia; IAPP, islet amyloid polypeptide (amylin); MAC, membrane attack complex (C5b-9); T2DM, type 2 diabetes mellitus. Created in BioRender. Zhao, J. (2026) https://BioRender.com/88y15uj.

C5a contributes to thinning of the fibrous cap and plaque rupture by inducing smooth muscle cell proliferation and migration as well as matrix metalloproteinase secretion, which are key steps leading to plaque instability and acute cardiovascular events. As a potent chemotactic factor and inflammatory mediator generated by complement activation, C5a and its receptor C5aR1 are expressed on multiple cell types within atherosclerotic plaques, including smooth muscle cells, macrophages, and endothelial cells. Upon binding to C5aR1, C5a activates multiple signaling pathways, promoting the transition of vascular smooth muscle cells from a contractile to a synthetic phenotype and enhancing their proliferative and migratory capacity [114]. Simultaneously, C5a stimulates smooth muscle cells and macrophages to secrete matrix metalloproteinases (MMPs), particularly MMP-2 and MMP-9, which degrade collagen and extracellular matrix components in the fibrous cap, leading to cap thinning and increased plaque vulnerability [114]. In patients with rheumatoid arthritis, C5a levels are positively correlated with carotid intima-media thickness, further supporting the role of C5a in vascular remodeling [115] (Figure 2).

## 8. Therapeutic Strategies Targeting the Complement System and Future Perspectives

Significant progress has been made in the development of complement-targeted drugs, with several agents approved or currently in clinical trials, targeting different components of the complement system. C5 inhibitors such as eculizumab and ravulizumab are the most widely used, blocking C5 cleavage to inhibit terminal complement pathway activation and thereby reducing the formation of C5b-9. Eculizumab, as the first approved complement inhibitor, has been successfully used to treat paroxysmal nocturnal hemoglobinuria (PNH) and atypical hemolytic uremic syndrome (aHUS) [116]. Ravulizumab, an improved version of eculizumab, extends the dosing interval from every 2 weeks to every 8 weeks by prolonging its half-life, significantly improving patient convenience, and has been shown to be non-inferior to eculizumab in multiple Phase III clinical trials [117,118]. In addition, the C3 inhibitor pegcetacoplan, by targeting C3, inhibits all complement activation pathways simultaneously and has demonstrated superior efficacy over C5 inhibitors in PNH patients, particularly in improving hemoglobin levels and reducing transfusion requirements [119,120]. The factor B inhibitor iptacopan, which inhibits the amplification loop of the alternative pathway, has also shown potential in PNH [121]. Complement-targeted drugs, owing to their potent immunomodulatory effects, exhibit unique advantages in the systemic treatment of metabolic diseases. For example, complement C3 also participates in the prothrombotic state in T2DM patients by affecting the fibrinolytic system [122,123]. Therefore, targeting the complement system may simultaneously ameliorate metabolic disturbances and inflammatory responses, offering new therapeutic strategies for T2DM and its complications. Table 1 shows key complement inhibitors with their targets, condition or disease, clinical phases and metabolic/renal endpoints.

The combination of complement-targeted therapy with conventional glucose-lowering drugs theoretically holds significant synergistic potential, enabling simultaneous improvement of metabolic control and inflammatory status. Metformin, a first-line drug for type 2 diabetes, primarily improves insulin sensitivity by activating the AMPK pathway, while GLP-1 receptor agonists lower blood glucose by promoting insulin secretion and inhibiting glucagon release. However, these drugs have limited effects on modulating chronic low-grade inflammation. Studies have shown that the complement system plays a central role in metabolic inflammation, with anaphylatoxins such as C3a and C5a promoting adipose tissue macrophage infiltration and insulin resistance [124]. Combining complement-targeted drugs (e.g., C5aR antagonists) with metformin may produce effects beyond those of monotherapy by simultaneously inhibiting complement-mediated inflammatory pathways and improving metabolic signaling.

However, although these drugs have shown good efficacy in complement-mediated kidney diseases and hematologic disorders, strategies targeting a single complement component alone may yield limited benefits in metabolic diseases. The pathogenesis of metabolic diseases such as T2DM and its complications involves multiple components of the complement system and a complex inflammatory network. For example, elevated complement C3 levels are closely associated with insulin resistance, fatty liver disease, and cardiovascular disease risk [106,125]. Furthermore, C5a promotes diabetic nephropathy progression by activating the STAT3 pathway and disrupting the gut–kidney axis [9]. Therefore, blocking only C5 or C3 may not fully suppress the excessive complement activation in metabolic inflammation and may overlook the pathogenic roles of other complement components such as C1q and MBL. For instance, low MBL genotypes are associated with an increased risk of cardiovascular events in T2DM patients, suggesting that the MBL pathway plays a distinct role in metabolic vascular complications [126].

Currently approved complement inhibitors are mostly administered systemically, which may increase the risk of immunosuppression, such as a higher risk of infections [127]. For lifelong complement inhibition in T2DM, the potential increased susceptibility to encapsulated bacterial infections, especially Neisseria meningitidis, necessitates vaccination and close monitoring, adding substantial complexity to chronic management [128]. Moreover, the high cost of chronic complement inhibitor therapy may impose a considerable economic burden and limit accessibility and adherence in the large T2DM population.

At the same time, future efforts should explore tissue-specific delivery systems tailored to metabolic tissues to maximize efficacy and minimize side effects associated with systemic immunosuppression. Concrete preclinical examples now support the feasibility of this approach. In the liver, GalNAc-conjugated siRNA targeting C3 decreased hepatic C3 by 96% [30]. In the kidney, lambda light chain–conjugated PLGA nanoparticles have been validated for megalin-mediated delivery to proximal tubule epithelial cells (PTECs), with specific retention for seven days and no renal toxicity after repeated dosing in mice; this platform could be adapted for local delivery of complement inhibitors [129]. In visceral fat, nanoparticles decorated with the prohibitin-binding peptide CKGGRAKEC or related adipose vasculature–homing peptides accumulate in white adipose tissue and have been used to deliver anti-inflammatory cargo, decreasing macrophage infiltration and improving insulin sensitivity in diet-induced obese mice [130]. These platforms provide a concrete basis for developing tissue-specific complement inhibitors. As our understanding of the mechanisms of the complement system in metabolic diseases deepens, these innovative strategies will open new avenues for the treatment of T2DM and its complications.

## 9. Conclusions

The role of the complement system in T2DM and its complications has expanded from traditional immune defense functions to a central hub of metabolic regulation—a paradigm shift that marks a deeper molecular understanding of T2DM pathophysiology. Based on current evidence, complement activation products do not simply promote or inhibit disease progression in a unidirectional manner; rather, they exert bidirectional regulatory effects across multiple dimensions, including insulin resistance, β-cell dysfunction, inflammasome activation, and tissue fibrosis, in a dose-dependent, tissue-specific, and cell-type-dependent manner. For instance, C3a and C5a facilitate inflammatory clearance in the acute phase but exacerbate tissue damage under chronic metabolic stress, while local expression of C1q and mannose-binding lectin in adipose tissue exhibits a non-linear association with insulin sensitivity. This complexity necessitates that, when interpreting findings from different studies, we distinguish between circulating complement levels and local tissue complement activation, and consider the interactive feedback between the complement system, insulin signaling, lipid metabolism, and the gut microbiota. From a translational perspective, complement components hold significant potential as biomarkers. However, fluctuations in a single complement indicator often lack specificity. Future efforts should focus on constructing combined models of multi-component complement profiles and metabolic parameters to improve the accuracy of T2DM risk stratification and prediction of treatment response. In terms of therapeutic strategies, inhibitors targeting the C5a receptor or C3 have shown promise in animal models for improving insulin resistance and delaying nephropathy progression. Nevertheless, caution is warranted regarding the potential risks of infection and negative impacts on tissue repair associated with long-term complement inhibition. Therefore, future research should prioritize the exploration of locally targeted delivery systems or intermittent dosing regimens to balance efficacy and safety. Ultimately, a deep dissection of the complement–metabolic network will drive the transition of T2DM management from a unified approach based on glycemic control toward precision interventions guided by immunometabolic subtypes. This will require the integration of multi-omics data, dynamic monitoring of complement activation status, and the inclusion of complement-related endpoints in clinical trials, thereby achieving a true translational loop from mechanism to clinic.

## Figures and Tables

**Table 1:** Key complement inhibitors with their targets, condition or disease, clinical phases and metabolic/renal endpoints.

Name	Target	ClinicalTrials.gov Identifier	Condition or Disease	Clinical Phase	Metabolic/Renal Endpoint
Eculizumab	C5	NCT06673394	Neuromyelitis Optica Spectrum Disorder Attack	Phase 2	None
C1 inhibitor	C1	NCT04489160	Traumatic Brain Injury, Trauma, Head	Phase 2	None
CE1145	C1 Esterase	NCT00292981	Hereditary Angioedema	Phase 3	None
Cinryze	C1 Esterase	NCT06359782	Subarachnoid Hemorrhage, Aneurysmal	Phase 2	None
Berinert	C1	NCT01134510	Kidney Transplantation	Phase 1 Phase 2	Serum creatinine; estimated GFR/eGFR; Deterioration of allograft function; Kidney biopsy evidence: capillaritis/inflammation/C4d deposition; Episodes of acute rejection/AMR
C1 esterase inhibitor	C1 Esterase	NCT02316353	Hereditary Angioedema Types I and II	Phase 3	None
Berinert P	C1 Esterase	NCT00748202	Hereditary Angioedema	Phase 3	None
C1 esterase inhibitor	C1 Esterase	NCT03221842	Refractory Antibody-Mediated Rejection (AMR) in Adult Renal Transplant Recipients	Phase 3	Decline in Estimated Glomerular Filtration Rate (eGFR)
C1 esterase inhibitor	C1 Esterase	NCT00785018	Endotoxemia, Inflammation, Multi-organ Dysfunction Syndrome	Not Applicable	Markers of Renal Injury
CINRYZE	C1 Esterase	NCT01759602	Neuromyelitis Optica	Phase 1	None
CE1145	C1 Esterase	NCT00168103	Hereditary Angioedema	Phase 2 Phase 3	None
C1 esterase inhibitor	C1 Esterase	NCT01766414	Innate Immune Response, Endotoxemia, Inflammation, Sepsis	Phase 3	None
C1 inhibitor	C1	NCT03051698	Asthma	Phase 4	None
C1 esterase inhibitor	C1 Esterase	NCT01147302	Graft Rejection	Phase 2	None
Eculizumab	C5	NCT06455709	Myasthenia Gravis, Generalized	Observational	None
Berinert	C1 Esterase	NCT02134314	End Stage Renal Disease, Kidney Failure, Delayed Graft Function, Ischemic Reperfusion Injury	Phase 1 Phase 2	Serum Creatinine; Creatinine Clearance
C1 esterase inhibitor	C1 Esterase	NCT01275976	Trauma, Inflammation, Sepsis, Multiple Organ Dysfunction Syndrome	Phase 3	None
Eculizumab	C5	NCT02727608	Diabetes Mellitus	Phase 2	Estimated glomerular filtration rate (GFR)
rhC1INH	C1	NCT02247739	Hereditary Angioedema	Phase 2	None
Danicopan	C5	NCT04469465	Paroxysmal Nocturnal Hemoglobinuria	Phase 3	None
RTB-021	C1 Esterase	NCT06919003	Kidney Transplant	Phase 2	Estimated Glomerular Filtration Rate
C1INH-nf	C1 Esterase	NCT01005888	Hereditary Angioedema	Phase 3	None
AMY-101	C3	NCT03694444	Gingivitis	Phase 1 Phase 2	None
AMY-101	C3	NCT03316521	Complement Mediated Diseases	Phase 1	None
Zimura	C5	NCT04435366	Geographic Atrophy, Macular Degeneration	Phase 3	None
AMY-101	C3	NCT04395456	Acute Respiratory Distress Syndrome Due to SARS-CoV-2 Infection (Severe COVID-19)	Phase 2	None
Soliris	C5	NCT04288713	Coronavirus	None	None
TP10	Complement Inhibitor	NCT00082121	Myocardial Ischemia, Coronary Arteriosclerosis, Aortic Valve Insufficiency, Mitral Valve Insufficiency	Phase 2	None
Pegcetacoplan	C3	NCT05809531	C3 Glomerulopathy or Immune-Complex Membranoproliferative Glomerulonephritis (VALE)	Phase 3	Urine protein-to-creatinine ratio (uPCR)
Pegcetacoplan	C3	NCT04579666	Amyotrophic Lateral Sclerosis, Motor Neuron Disease	Phase 2	None
Eculizumab	C5	NCT05886244	Paroxysmal Nocturnal Hemoglobinuria	Phase 3	Serum LDH
ZIMURA	C5	NCT03374670	Idiopathic Polypoidal Choroidal Vasculopathy	Phase 2	None
Conestat Alfa	C1 Esterase	NCT04414631	Coronavirus Infections	Phase 2	None

## Data Availability

No new data were created or analyzed in this study. Data sharing is not applicable to this article.
